# Reoperation Rates Following Lumbar Decompression Surgery at Three or More Levels

**DOI:** 10.1007/s43465-025-01673-2

**Published:** 2026-01-05

**Authors:** Ryan J. Hoel, Jason J. Haselhuhn, Melissa S. Albersheim, Sharon C. Yson, Jeffrey Thomas P. Luna, Paul Brian O. Soriano, Jonathan N. Sembrano, Kari Odland

**Affiliations:** 1 Twin Cities Orthopedics, 4010 W 65th Street, Edina, MN 55435 USA; 2 The Department of Orthopedic Surgery, University of Minnesota, 2512 South 7th Street, Suite R200, Minneapolis, MN 55455 USA; 3 Minneapolis Veterans Affairs Health Care System, One Veterans Drive, Minneapolis, MN 55417 USA

**Keywords:** Multilevel decompression, Laminectomy, Lumbar spinal stenosis, Reoperation rates

## Abstract

**Objective:**

In this retrospective study, we sought to investigate the reoperation rates of patients after multilevel (≥ 3) contiguous lumbar decompressions without concomitant fusion.

**Methods:**

We reviewed all spine surgeries performed over a 15-year period at a single Veterans Affairs hospital and identified patients who underwent multilevel lumbosacral (L4-S1) decompressions without fusion. Records were reviewed to confirm the surgery performed and determine if any subsequent spinal operations were performed. Patients with follow-up of less than 1 year were excluded.

**Results:**

Forty-five patients (44 M:1 F), with a mean age of 68 years, met minimum 1-year of follow-up (median 27 months) and were included in the final analysis. Three patients (7%) underwent subsequent fusion at a previously decompressed level at 14, 23, and 25 months. Another three (7%) underwent revision decompression at previously decompressed levels at 6, 24, and 130 months. Five patients (11%) underwent reoperation in the first 2 months for wound complications related to infection or hematoma. One patient (2%) underwent debridement of an epidural abscess located three levels cranial to the most proximal decompression 6 years after index surgery; this was determined to be unrelated to the prior decompression. In total, 12 patients (27%) underwent reoperation at time of final follow-up.

**Conclusions:**

The need for subsequent arthrodesis following decompressions of three or more consecutive levels without concomitant fusion may be less than intuitively suspected. Concomitant fusion at the time of index surgery is not necessarily warranted solely based on the number of levels decompressed.

## Introduction

Lumbar degeneration is a common cause of back pain for which patients seek care. While nonoperative management may provide symptom relief, some patients require surgical intervention. Lumbar decompressions can successfully reduce pain and improve the quality of life. [1–5]

The role of concomitant arthrodesis, particularly in multilevel decompressions, remains debated [6, 7]. Concerns about instability arise because decompression often involves the removal of posterior bony and ligamentous structures [8]. Preexisting spinal instability, or instability created during surgery, is thought to increase the likelihood of needing subsequent fusion, particularly in patients with increasing number of spinal levels included in the decompression. [1, 9, 10]

Conventional wisdom suggests that decompression of multiple should be combined with fusion at the same level(s) to prevent subsequent instability [1, 9, 11, 12]. However, concomitant fusion increases operative time, blood loss, and perioperative morbidity, and long-term reoperation risk [7, 9, 13–15]. Recent studies have challenged the assumption that multilevel decompression requires prophylactic fusion. Prior work has focused on fusion with one- to two-level decompressions [10, 16–20], with few studies examining outcomes following multilevel decompression of three or more spinal levels. In this retrospective study, we sought to investigate reoperation rates in patients undergoing multilevel (≥ 3) lumbar decompressions without fusion at a Veterans Affairs hospital.

## Methods

This single-center, retrospective study was approved by the Institutional Review Board. Since all data were deidentified, the study was considered minimal risk and granted an exemption from obtaining patient consents.

### Patient Selection

**Fig. 1:**
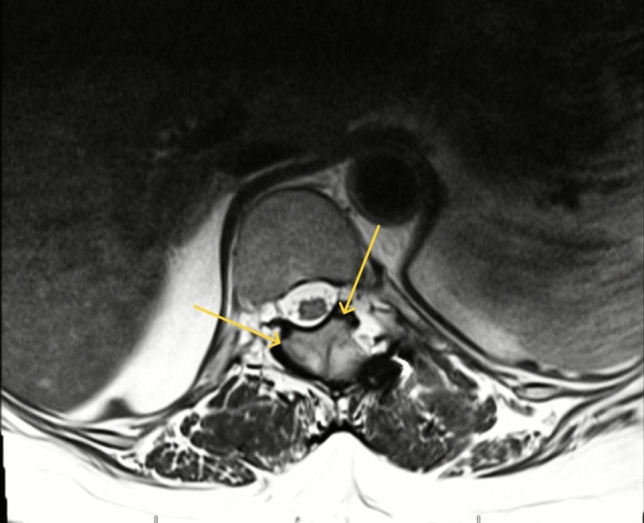
MRI demonstrating stenosis of the lumbar spinal cord

We reviewed the charts of all spine surgeries performed over a 15-year period at a single Veterans Affairs (VA) tertiary referral hospital. Patients who underwent multilevel (≥ 3) lumbosacral decompressions without concomitant fusion were identified. Necessity for multilevel lumbar decompression in these patients was determined based on assessment of physical exam and radiographic imaging, which reflected preoperative spinal instability. Spinal instability has been previously defined as excessive sagittal plane translation or rotation involving multiple spinal columns, causing painful motion [21]. Patients with < 1 year of follow-up were excluded. The follow-up clinic visits were reviewed to determine if any further spinal operations were performed in the VA system. To supplement our records, patients were contacted by phone regarding surgeries performed outside of our institution. If a patient was unable to be contacted after several attempts, the follow-up time point was recorded as the last clinic visit with our department. Demographics, levels of decompression, indications (symptomatic lumbar stenosis with claudication or radiculopathy) (Fig. 1), and all reoperations were recorded. Reoperation-free survival was analyzed using Kaplan–Meier estimates with 95% confidence intervals.

### Surgical Technique

All cases were done via an open posterior approach with a midline skin incision. Intervening interspinous ligaments were removed, while portions of the spinous processes were preserved when possible. In some cases, the lamina was completely removed; in others, a cranial “inverted U-shaped” portion of the caudal lamina was removed. In all cases, the bilateral pars interarticularis were preserved to reduce the risk of segmental instability. Bilateral medial facetectomies and foraminotomies were routinely performed at the decompressed levels. Adequate decompression of the nerves was confirmed with a probe. Hemostasis was achieved with cautery, andwounds were irrigated and closed wound in layers with sutures.

## Results

We initially identified 56 patients who met inclusion criteria, having undergone posterior decompressions at three or more consecutive levels. Of those, 45 (80%) had 1 year of follow-up and were included in the final analysis. Included were 44 males (98%) and 1 female (2%) with a mean age of 68 years. The median follow-up time of the 45 included patients was 27 months.

**Fig. 2:**
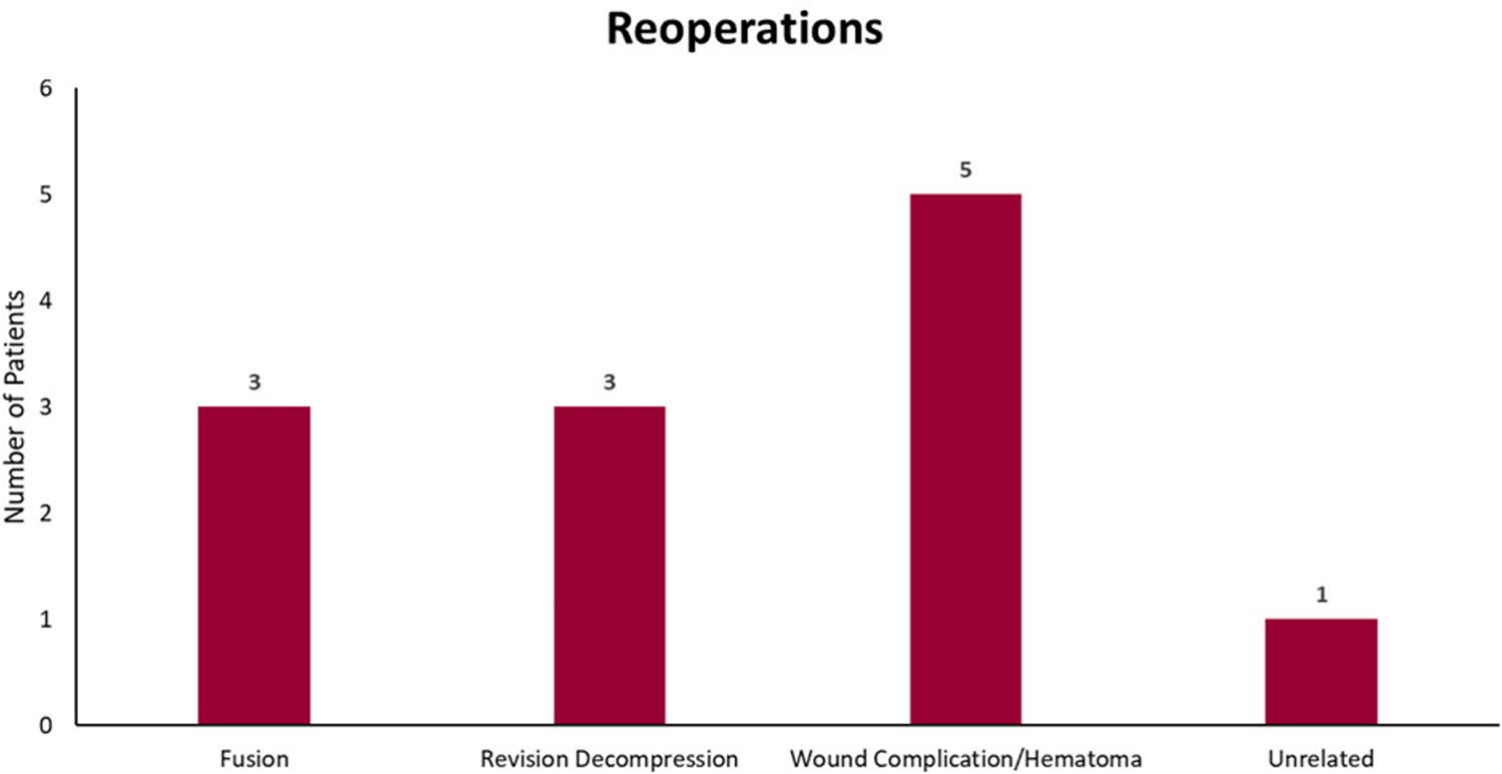
Number of reoperations by procedure (N = 45)

**Fig. 3:**
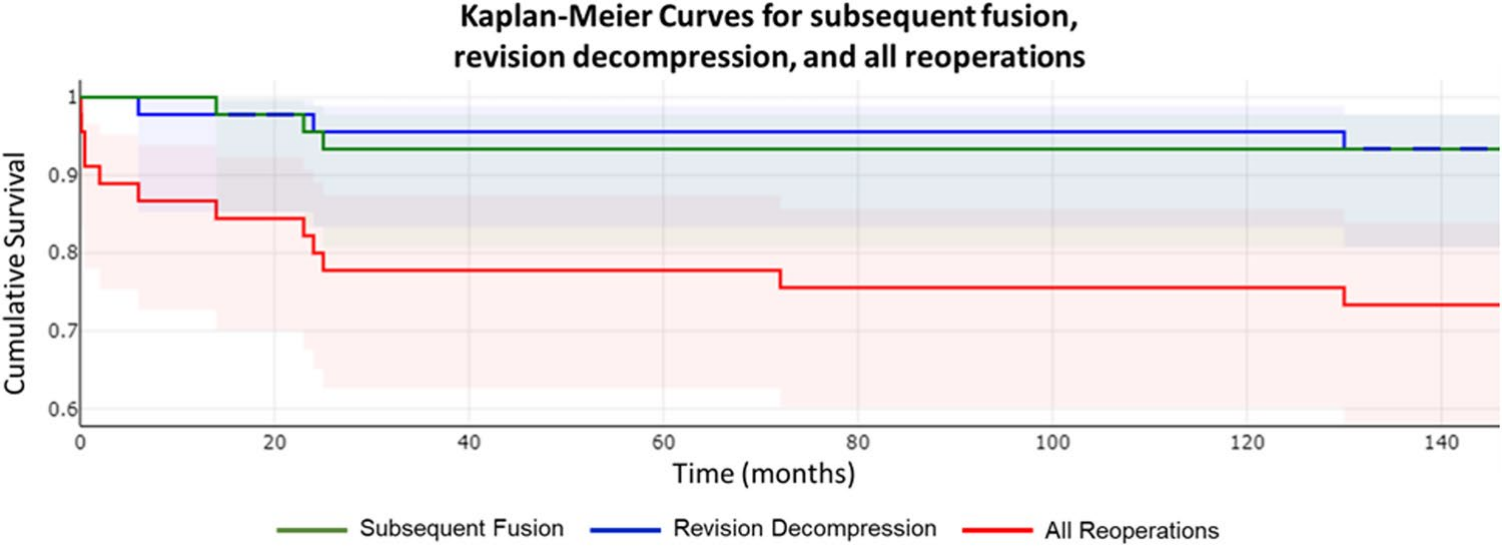
Kaplan–Meier curves for revision decompression, subsequent fusion, and all reoperations

Of the 45 patients included in final analysis, 6 went on to subsequent arthrodesis or decompression (14%) (Fig. 2). Three (7%) went on to subsequent arthrodesis at a previously decompressed level at 14, 23, and 25 months (Fig. 3), and another three patients (7%) underwent revision decompression at previously decompressed levels at 6, 24, and 130 months.

Five patients (11%) underwent early reoperation in the first 2 months for wound complications related to infection or hematoma. One patient (2%) underwent debridement of an epidural abscess located three levels cranial to the most proximal decompression 6 years after his index surgery; the abscess was determined to not be related to the prior decompression. In total, 12 (27%) patients underwent reoperation at time of final follow-up.

## Discussion

This retrospective study of a total of 56 patients analyzed reoperation rates following multilevel lumbar decompression without concomitant fusion, with particular interest in reoperations for subsequent fusion at previously decompressed levels. In patients with 1-year follow-up, there was an overall reoperation rate of 27%. However, the rate of subsequent fusion and revision decompression surgeries was relatively low. These findings suggest that routine prophylactic fusion for multilevel lumbar decompression is not always necessary, provided stabilizing structures are preserved and preoperative instability is absent. To our knowledge, this is the first study specifically investigating reoperation rates for any indication after traditional open multilevel (≥ 3) decompressions without fusion.

**Fig. 4:**
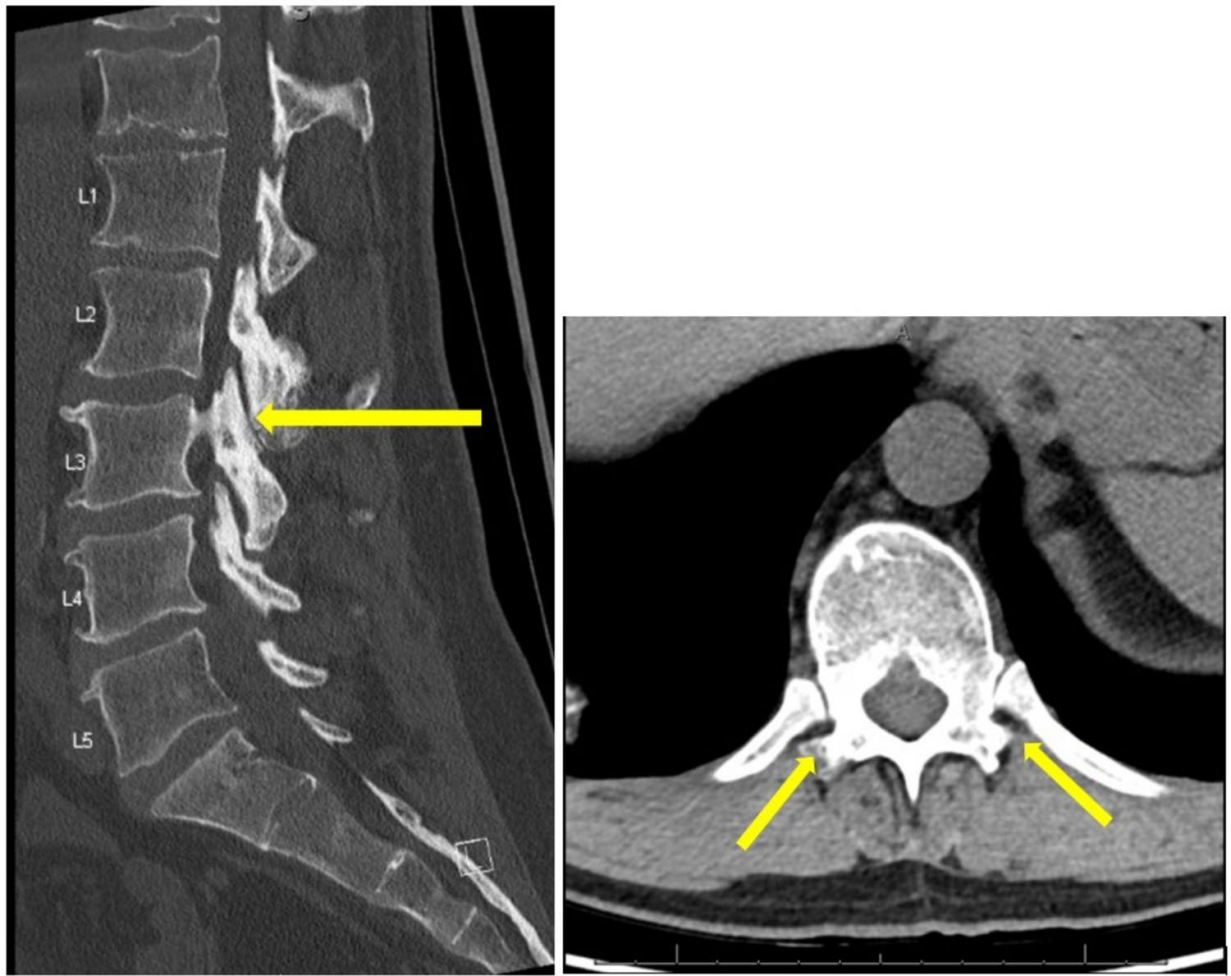
Low-dose computed tomography (CT) scan of the lumbar spine demonstrating bilateral pars at L3 with surrounding sclerosis (yellow arrows)

The incidence of subsequent fusion during early follow-up of these patients was relatively low, with 7% of patients undergoing fusion at a mean of 21 months. These findings are similar to those of Nolte et al. [22], who retrospectively reviewed 32 patients who underwent multilevel (≥ 3) primary lumbar laminectomy without fusion using a stability-preserving technique and found 2 patients (6.2%) underwent a revision to a fusion at a mean of 25.4 months. There was no mention of reoperations for wound or hematoma complications, but they did report no significant difference in the likelihood of reoperation between those who had single-level versus multilevel laminectomies (9.43% v. 6.45%) at a mean follow-up of 24.2 months (OR = 4.46, p = 0.234). The surgical technique in both studies preserved the bilateral pars interarticularis, which helps maintain segmental stability and may lower the risk of the need for subsequent fusion (Fig. 4). Together, these studies show that decompression of three or more levels without concomitant fusion can be efficacious without imparting a high risk of the need for subsequent fusion.

Prior studies have considered multilevel decompression to be two or more levels. Although the biomechanical changes that result from decompression of additional levels may influence outcomes, these studies provide a baseline to compare against decompression of three or more levels. Adilay et al. [23] reviewed 64 multilevel decompression patients (50 two-level and 14 three-level) and found 4 (6.25%) required subsequent posterior instrumented fusion due to postoperative spondylolisthesis at a mean follow-up of 42.8 months. However, they did not specify if these were two-level or three-level laminectomy patients. The authors used a posterior approach with a midline incision and preserved the facet joints, similar to our study, which helps maintain stability while allowing adequate access to the spine.

In contrast, Khanna et al [24]. reported a high rate of subsequent fusion in their review of 92 multilevel (2–4 levels) minimally invasive laminectomy and/or microdiscectomy patients, of which 23 were ≥ 3 levels. Of these, five patients (21.7%) required a subsequent lumbar fusion, three for persistent/worsening foraminal stenosis, and two for development of grade one L4-5 spondylolisthesis. They employed a unilateral approach through a paramedian incision to perform minimally invasive laminectomies, which allowed an 18–22 mm diameter tubular working channel to be used. While this technique may result in less soft tissue disruption than a traditional open approach, we hypothesize it may have contributed to the three patients developing persistent/worsening foraminal stenoses.

A recent study by Yamamoto et al. [25] compared 2-year outcomes for patients who underwent single-level versus multilevel (≥ 3) decompression using a lumbar spinous process-splitting laminectomy, which was first described by Watanabe et al. [26] In this technique, the spinous process is split in half longitudinally, and the paraspinal muscles are left attached to the lateral aspects bilaterally. After decompression is complete, the spinous process halves are reapproximated using suture. The proposed benefits of this technique are minimal damage to the paraspinal muscles and preservation of the supraspinous ligaments, interspinous ligaments, and spinous process. Five of 122 patients (4.1%) in their multilevel decompression cohort required revision surgery within 2 weeks of index surgery; however, they did not report what procedures were performed. In contrast, none of our patients required revision decompression or subsequent fusion within 2 weeks.

Another interesting finding from our study was the high rate of early reoperations due to wound complications (11%) related to infection or hematoma. This was similar to Adilay et al. [23], who described four infections and two hematomas in their multilevel decompression cohort (9.4%), although they did not specify if these patients received a two- or three-level decompression. Similarly, Ulrich et al. [27] reported wound/osseous infections in five patients (4.6%) and complications including urosepsis, hemorrhage, or wound-healing deficits in 15 patients (13.9%) in their multilevel (≥ 2) decompression without concomitant fusion cohort. Yamamoto et al. [25] reported perioperative complications including surgical site infection, extradural hematoma, or spinal fluid leak occurred in ten multilevel decompression patients (8.2%). They also found this rate did not differ significantly from patients who underwent single-level decompression. Despite the differences in decompression techniques, our results are similar, which suggests these complications occur at a relatively low rate after decompression of three or more levels. Our findings contrast with Khanna et al. [24], who had no surgical site infections (0%) and one epidural hematoma (1.1%) that required reoperation on postoperative day three. We suspect these low rates are due to their minimally invasive approach as described above and their patient population.

It has been reported that veterans have poorer overall health and higher rates of medical comorbidities than the general population due to combat-related physical and psychological conditions [28], which may help explain the relatively high rate of wound-related problems and early reoperation rate in our cohort.

Our study did have several limitations. Most notable is the lack of patient-reported outcomes data at the preoperative and postoperative time points. Furthermore, when obtaining supplement records from patients over the phone, we recognized that this may have introduced recall bias into some of our follow-up data. In addition, we have a relatively short follow-up, with the median follow-up being 27 months. We also acknowledge this is a relatively small sample size and the patients in this cohort were all veterans, which limits the generalizability of our findings. However, we reviewed all cases during the study period in an attempt to minimize selection bias. Lastly, this study analyzed only patients with ≥ 3 level decompression and did not include a comparator group. Future studies with propensity-matched controls could provide further insight into all-cause reoperation rates after multilevel lumbar decompression without concomitant fusion.

## Conclusion

In conclusion, our results suggest that the need for subsequent fusion after decompressions of three or more consecutive levels without concomitant fusion may be less than dictated by conventional wisdom and broad clinical practice. The necessity for concomitant fusion should be determined on a case-by-case basis as opposed to the basis of the number of levels decompressed to adequately relieve patient symptoms. There is a need for further investigation on the mid-term and long-term rates of subsequent fusion after these high-risk decompressions. This study lends information for preoperative discussion with patients considering the risks and benefits of undergoing decompression compared to fusion.

## Declarations

### Availability of data and materials

The original contributions presented in this study are included in the article. Further inquiries can be directed to the corresponding author(s).
